# Thinking outside the boxes: analyzing the current landscape of popular behavioral tests for adult zebrafish

**DOI:** 10.1038/s41684-026-01694-w

**Published:** 2026-02-26

**Authors:** Allan V. Kalueff, Adam Michael Stewart, Murilo S. de Abreu, Matthew O. Parker

**Affiliations:** 1 Department of Biosciences and Bioinformatics, School of Science, Xi’an Jiaotong-Liverpool University, Suzhou, China; 2 Suzhou Municipal Key Laboratory of Neurobiology and Cell Signaling, School of Science, Xi’an Jiaotong-Liverpool University, Suzhou, China; 3 The International Zebrafish Neuroscience Research Consortium (ZNRC), New Orleans, LA USA; 4 Institute of Translational Biomedicine, St. Petersburg State University, St. Petersburg, Russia; 5 Graduate Program in Health Sciences, Federal University of Health Sciences of Porto Alegre, Porto Alegre, Brazil; 6 Surrey Sleep Research Centre, School of Biosciences, University of Surrey, Guildford, UK

**Keywords:** Neuroscience, Scientific community

## Abstract

Zebrafish are commonly tested in various behavioral assays. To better understand such practices, we surveyed active global zebrafish neuroscience labs, asking to rank such assays based on their perceived utility. Overreliance on few well-established assays and their inconsistent nomenclature call for further innovation and standardizing of adult zebrafish neurobehavioral testing.

Zebrafish (*Danio rerio*) have rapidly become a widely used model organism in biomedical research and drug discovery, owing to their high genetic and physiological homology with humans^1^. Their translational utility is especially evident in behavioral neuroscience^2^, with the number of Pubmed items on zebrafish brain and behavior doubling in the past decade. Zebrafish are increasingly utilized to model brain disorders^3^ and study complex behaviors^4,5^. However, this research depends critically on providing appropriate testing conditions^6^, accurate identification of relevant behaviors^7,8^, the use of validated protocols^9^, high resolution of behavioral tracking^10^, robust data interpretation^11^ and reproducibility of findings across and within laboratories^12,13^.

There has been a recent growing effort to optimize zebrafish neurobehavioral research, including testing different housing environments and procedures^14^, as well as behavioral endpoints^7^. However, these assays themselves represent a critical bottleneck. It is therefore timely to assess the current landscape of adult zebrafish behavioral assays and to identify the limitations and opportunities for its advancement.

## Current landscape

To better understand practices and stimulate discussion around behavioral assay use, the International Zebrafish Neuroscience Consortium (ZNRC) has conducted a targeted survey, which was distributed in June 2025 to nearly 50 active zebrafish laboratories worldwide, both members and non-members. The survey posed a single question, asking laboratories “to list and rank their top 10 adult zebrafish behavioral assays by a general utility for the field”, with a specific request to focus on individual behavioral tests (e.g., novel tank, mirror test), rather than broader constructs (e.g., anxiety/fear) or contexts (e.g., predator exposure). The results were collected 4 months later and analyzed, scoring the frequencies of mentioning each test (and its respective rank, from 1 to 10), expressed as % of total number of responders for each rank.

Overall, twenty active laboratories responded, offering a snapshot of commonly used assays and highlighting both expected trends and less conventional choices (Table 1). Nearly 80% responders identified the novel tank test as the most useful behavioral assay in adult zebrafish, with another 16% listing it as the second most useful test. The open field test was ranked second by 35%, followed by the light-dark test (11%). Other assays frequently appearing in the top five include the T/Y-maze and the shoaling tests (16% each). The shoaling test was listed as the fourth most useful by 22% responders, followed by the T/Y-maze and the social preference test (11% each). The fifth position in the ranking was shared by the mirror exposure test, the light-dark box test and the T/Y-maze (11% each). Cumulative analyses of these and other tests are summarized in Table 1.

**Table 1:** Top most useful adult zebrafish behavioral assays ranked by active zebrafish laboratories responding to the survey

Behavioral assay	Test specifics	Main targeted domains	Number of responders
The novel tank test	Assesses the natural tendency for protective bottom swimming (geotaxis), initially avoiding a more aversive, top area of an unfamiliar tank. Over time, fish gradually habituate and begin to explore the top areas of the tank.	Locomotion and novelty-evoked anxiety	17
The open field test	Evaluates spontaneous locomotor activity and protective peripheral (thigmotaxis) vs. more aversive ‘central’ swimming in a novel arena.	Locomotion and novelty-evoked anxiety	10
The light-dark box test	Examines the innate aversion of adult zebrafish to brightly lit area (vs. more protective dark area, scototaxis) in the two-chamber box with the lit and dark halves.	Novelty-evoked anxiety	10
T/Y-maze	Tests spatial learning and memory, as well as spontaneous alternation in the free-swimming version; can also be sensitive to behavioral perseverations	Spatial memory, repetitive behaviors	9
The shoaling test	Examines zebrafish group behavior by assessing their shoal parameters (size, average inter-fish distance, polarity)	Group (shoaling) behavior	8
The social preference test	Evaluates the preference of fish for social interaction with conspecifics over empty compartment or another stimulus	Social interest	6
The mirror exposure test	Assesses zebrafish aggression by attacking their own reflection in the mirror	Mirror-evoked aggression	3
The novel object test	Evaluates novelty recognition by comparing preference for unfamiliar vs. familiar object	Novelty recognition	2
Conditioned place preference	Assesses the rewarding effects of a stimulus by measuring zebrafish preference for the environment associated with it	Conditioning	2
Conditioned fear test	Evaluates the ability of zebrafish to associate (‘condition’) a neutral stimulus with an aversive event	Conditioning	1

Results based on responses from 20 laboratories.

Other tests listed among top 5 by the responders included the plus maze test, conditioned aversion test and the zebrafish tail immobilization (ZTI) test. Tests ranked 6-10, based on their utility, included the social preference and the novel object tests (33% each), the T/Y-maze and mirror test (28% each), the shoaling test and the open field test (22% each), the ZTI test and conditioned place preference (CPP) (16% each), as well as conditioned place aversion test and dyadic aggression test (6% each). Less frequently used tests, mostly ranked as 6-10 in the list, included the inhibitory avoidance test (4 labs), the plus-maze test (2 labs) and the 5-choice serial testing test (1 lab). Three unclear entries involved aggression display, aggression and swimming test. The predator exposure test was mentioned twice and was excluded due to its ‘context’ nature (i.e., listed as an experimental manipulation in a well-established test rather than a test on its own). Overall, this informal audit reveals widespread reliance on a small core of broadly applicable tests, alongside more domain-specific assays used by subsets of laboratories. Given the field’s heavy reliance on novelty-based behavioral tests (Table 1), this also raises important questions about the breadth, diversity and conceptual foundations of behavioral tools currently in use across the field.

## Potential implications

These survey results (Table 1) suggest that the field is dominated by a few widely used, general-purpose behavioral assays (novel tank test > open field, light-dark box > T/Y-maze and shoaling test), and several domain-specific assays. This likely reflects their broad applicability, relative ease of use, and sensitivity to pharmacological, genetic and environmental manipulations. However, their ubiquity also highlights a lack of diversity in behavioral paradigms, which may constrain the range of the central nervous system (CNS) domains explored in zebrafish research.

Notably, the novel tank test stands out as the clear front-runner. Its popularity is understandable, as the test offers a rapid, single-trial measure of locomotion and anxiety-like behavior and is highly sensitive to a wide array of experimental manipulations^14,15^. However, its broad scope (capturing activity, neophobia, freezing, exploration and even social behavior) complicates interpretation. Furthermore, its multidimensionality may also hinder reproducibility across labs, given the many intrinsic (e.g., personality, sociality and stress responsiveness) and extrinsic (e.g., lighting, tank geometry and water characteristics) factors that influence outcomes^14^. In this sense, the novel tank test exemplifies both the utility and the interpretative challenge of multi-domain assays^11^.

Interestingly, the ranking of top aquatic behavioral models shows a strong emphasis on locomotor, anxiety-like, social and cognitive phenotypes, aligning with current trends in zebrafish CNS disease modeling^5^. However, we also note a clear absence of more innovative or recently developed useful assays (e.g., the ZTI test or the 5-choice serial testing test) in the top rankings. This may suggest a field that is methodologically conservative and relying on a small toolbox rather than exploring conceptually novel behavioral paradigms. Indeed, although more than 20 distinct assays were mentioned in total, this is very modest compared to the much larger number of behavioral tools available for rodents. The latter aspect again calls for further innovative studies of zebrafish behavior and the development of novel assays based on both new concepts and newly recognized behavioral phenomena^8^, as well as targeting disorders presently underrepresented in zebrafish CNS disease models^5^.

Another notable observation is the widespread adaptation of rodent-derived tests (e.g., the open field and the light-dark box assays), with relatively few paradigms rooted in fish-specific behaviors or ethology. Aside from the shoaling and mirror tests, which draw on naturalistic social and aggressive interactions, most listed assays rely on artificial environments and ‘forced’ tasks. While this may enhance translatability across species, it also risks neglecting ecologically relevant behaviors that might yield new insights. As such, there is a clear opportunity here for the development of assays that better reflect zebrafish biology and natural behavioral repertoires (see^9^ for review).

Finally, the survey has revealed substantial inconsistency in terminology, with multiple names used to describe the same assays (e.g., “novel tank test,” “novel tank diving test” and “novel tank dive task”). This lack of standardization hampers literature searches, systematic reviews and meta-analyses, likely reflecting a relatively early stage of the field’s methodological consolidation.

## The way forward: how to build innovation?

Several technical and financial barriers in creating and validating a new behavioral assay may be one of the reasons why this important line of research is presently stifling. First, there are limited incentives, especially outside industry, for developing novel zebrafish behavioral assays, given the time, cost and effort required to validate them. Second, demands for rigorous standardization and reproducibility^12,13^, while valuable, may at the same time discourage exploratory assay development. Finally, there remains little infrastructure for systematic cross-laboratory validation of new tests, making it harder to establish credibility for novel paradigms.

Expanding the zebrafish behavioral repertoire will require both conceptual and practical changes (Box 1). Conceptually, there is a need to move beyond general-purpose assays toward tests that capture underexplored domains and disorders^3^ (e.g., personality disorders or pathological hyper-sociality), including those not easily modeled in rodents. Ethologically grounded and species-specific assays (e.g., predator- or alarm cue exposure-based), capitalizing on zebrafish naturalistic or free-ranging behavior, sensory ecology and decision-making, represent another potentially promising direction. The current focus on forced-exposure paradigms (e.g., confinement to novel and/or anxiogenic tanks) can be complemented by “free-choice” or home-tank-based tests that capture spontaneous curiosity, social negotiation or place learning: for instance, simply removing a divider in a large tank to reveal unexplored space can elicit robust exploratory behavior without evoking stress-like bottom-dwelling responses (see^16^).

Another opportunity lies in the design of ‘hybrid’ assays that combine the elements of multiple tasks into a single paradigm, such as maze-based exploration followed by social or novelty preference testing. This approach, already common in rodent laboratories, can improve efficiency and allow richer behavioral phenotyping. Complementing this, the use of artificial intelligence (AI) tools, such as powerful open-source DeepLabCut and SimBA (Simple Behavioral Analysis) platforms, have the capacity to automate the classification of animal behaviors, enabling higher-dimensional behavioral analyses that go beyond traditional endpoints like “distance moved” or “time spent freezing”. These tools can facilitate the discovery of previously unquantified behaviors, improve cross-laboratory consistency and help unlock the full complexity of zebrafish behavioral repertoires.

The noted presence of terminological discrepancies in zebrafish literature also suggests that in addition to community efforts to standardize zebrafish behavioral descriptions^7,8^, special effort may also be needed to develop a more consistent and simplified description of behavioral assays per se, to ensure data compatibility and comparability (Table 2). If successful, this effort will not only assist the newly established labs to navigate the existing behavioral literature but will also help improve data searchability in the literature, thereby streamlining and facilitating systematic review and meta-analysis processes.

**Table 2:** Proposed uniform classification of selected popular adult zebrafish behavioral tests

Proposed test name and abbreviation	Synonyms seen in research literature or used in the ZNRC survey
The novel tank test (NTT)	The novel tank, novel tank diving test, novel tank dive test/task/assay/paradigm, top-bottom preference, novelty tank test, novel aquarium test
The light-dark test (LDT)	The light-dark box, dark-light test (box), black-white [tank] test/task/assay/paradigm, dark-white preference test, black-white shuttle box/test
The open field test (OFT)	The open field, open tank (including modifications), open arena test, open novelty test/assay/task/paradigm
The shoaling test (ST)	The shoal test/assay, shoal formation test, shoal cohesion test/task, shoaling behavior test, schooling test
The T/Y-maze [test] (TMT, YMT)	The T/Y-shaped maze [test/task] (including the free movement pattern versions)
The novel object test (NOT)	The novel object recognition test/task/assay/paradigm, novel object exposure test, novel object discrimination test/task
The mirror test (MT)	The mirror-biting test/task/assay/paradigm, mirror-induced aggression test, mirror exposure test, mirror aggression test
The social preference test (SPT)*	Social preference, social preference task/assay/paradigm, social interaction test, social investigation test/task
The social interaction test (SIT)**	Social interaction test/task/assay/paradigm, social investigation test, dyadic interaction test
The social recognition test (SRT)***	Social interaction test/task/assay/paradigm
Conditioned place aversion (CPA)****	Conditioned place avoidance, CPA test/task/paradigm
Conditioned place preference (CPP)	Conditioned placement preference, CPP test/task/paradigm
The Zebrafish tail immobilization (ZTI) test	-

*refers to preference for conspecific(s), but is often used as a synonym of SIT.

**refers to dyadic interactions, but is often called SPT.

***refers to the ability to recognize familiar vs. unfamiliar conspecific. ****differs from the conditioned fear test (CFT, fear conditioning test/assay/paradigm) that typically measures involuntary responses like "freezing" (immobility) in response to a cue (tone) or context associated with an aversive stimulus (e.g., electric shock)

Box 1 Summary of potential solutions to facilitate innovation in zebrafish behavioral assays

**Table:** 

**Conceptual**
• Identify and target novel zebrafish behaviors
• Target novel behavioral domains or syndromes currently underrepresented in zebrafish literature
• Develop ‘hybrid’ assays that simultaneously target several behavioral domains, or ethologically meaningful test batteries
• Apply novel concepts (e.g., free exploratory vs. ‘forced’ novelty paradigms)
• Apply evidence from zebrafish behavioral biology and ecology, target more fish-specific behaviors
**Practical**
• Facilitate cross-laboratory validation
• Continue to standardize behavioral terminology (ethograms)
• Integrate behavioral assays with modern artificial intelligence (AI) and deep-learning tools to enhance behavioral testing
• Utilize virtual reality (VR) tools to standardize and enhance behavioral testing in zebrafish
**Additional**
• Standardize housing conditions and experimental protocols and their description
• Standardize terminology related to behavioral assays (see Table 2 for details)

## Conclusion

To move forward, we need to ‘think beyond the current zebrafish behavioral boxes’, not only in terms of apparatus design, but also in how we conceptualize behavior and its role in modeling brain function. Most existing assays are built narrowly on the assumptions that behavior can be reduced to discrete endpoints; that stress or novelty exposure is the most tractable way to reveal phenotypes; and that rodent paradigms provide the default ‘blueprint’ for cross-species modeling. Although these assumptions have been useful to get us to the current point, they limit the field’s ability to explore more dynamic, integrative or ecologically grounded forms of behavior. Richer, more informative assays will emerge if we treat behavior as a systems-level output, shaped by perception, internal state, memory and social context, rather than as a set of isolated responses. This shift would align with emerging frameworks in computational and systems neuroscience, including active inference^17^ or embodied cognition^18^, both of which emphasize the interplay between motivation, learning and exploration. Furthermore, zebrafish are amenable to whole-brain imaging, genetic manipulation and high-throughput testing^1^, making them uniquely positioned to support this kind of integrated behavioral neuroscience if the behavioral tools can evolve accordingly.

To achieve this, we will need to develop assays that capture underused domains, accommodate more natural, free-ranging behaviors and make better use of digital tools for behavioral quantification or modulation. This will also require a cultural shift: we must push towards exploratory and ethologically grounded assay development as a legitimate and necessary scientific endeavor, not just a methodological ‘side show’. In conclusion, conceptual innovation in behavioral testing is central to model system utility and translatability.
